# Supplementary material for: DrugDevCovid19: An Atlas of Anti-COVID-19 Compounds Derived by Computer-Aided Drug Design
Source: Molecules. 2022 Jan 21;27(3):683. doi: 10.3390/molecules27030683 (PMC8838031; doi:10.3390/molecules27030683)
Supplement: Supplementary file 1 [file molecules-27-00683-s001.zip › Table S3.pdf]

Table S3 The recommended compounds against Mpro of SARS-CoV-2 with activity assay in the corresponding CADD study.

Candidates	Groups	Researchers	DOI	PubChem CID	Drugbank ID	Activity	Docking Score
**Telaprevir**	Approved, Withdrawn	Pathak et al.	10.1021/acsnano.0c07383	3010818	DB05521	IC50=11.47μM	-8.4
**Efonidipine**	Experimental	Mohammad et al.	10.1021/acsmedchemlett.0c00521	119171	DB09235	IC50=38.5 μM	-8.3
**Nelfinavir**	Approved	Pathak et al.	10.1021/acsnano.0c07383	64143	DB00220	IC50>10μM;EC50=3.28μM	-8.3
**Manidipine**	Approved, Investigational	Mohammad et al.	10.1021/acsmedchemlett.0c00521	4008	DB09238	IC50=4.8 μM	-7.8
**Epigallocatechin-3-gallate (EGCG)**	Investigational	Du et al.	10.1016/j.ijbiomac.2021.02.012	65064	DB12116	IC50=0.874 μM	-7.8
**Bedaquiline**	Approved	Mohammad et al.	10.1021/acsmedchemlett.0c00521	5388906	DB08903	IC50=18.7 μM	-7.7
**Dipyridamole**	Approved	Liu et al.	10.1016/j.apsb.2020.04.008	3108	DB00975	IC50=0.53 μM;EC50=100 nM	-7.4
**Lercanidipine**	Approved, Investigational	Mohammad et al.	10.1021/acsmedchemlett.0c00521	65866	DB00528	IC50=16.2 μM	-7.3
**Boceprevir**	Approved,	Mohammad et al.	10.1021/acsmedche	10324367	DB08873	IC50=5.4 μM	-7.2

	Withdrawn		mlett.0c00521				
**Boceprevir**	Approved, Withdrawn	Pathak et al.	10.1021/acsnano.0c07383	10324367	DB08873	IC50=1.42µM;EC50=49.89µM	-7.2
**Cinanserin**	Drug-like	Jin et al.	10.1038/s41586-020-2223-y	5475158	/	IC50=125 µM	-6.7
