# Supplementary material for: DrugDevCovid19: An Atlas of Anti-COVID-19 Compounds Derived by Computer-Aided Drug Design
Source: Molecules. 2022 Jan 21;27(3):683. doi: 10.3390/molecules27030683 (PMC8838031; doi:10.3390/molecules27030683)
Supplement: Supplementary file 1 [file molecules-27-00683-s001.zip › Table S5.pdf]

Table S5. The CADD compounds with high structural similarity to the Mpro inhibitors.

PubChem CID	Compounds structure	Compounds Name	Groups	Inhibitor	FitDock
**9871747**	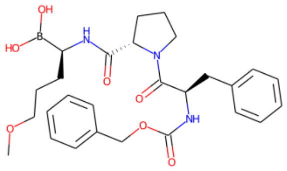	Flovagatran	Investigational	MPI1	*Due to the unusual B atom in the compound, FitDock cannot recognize it*
**23304231**	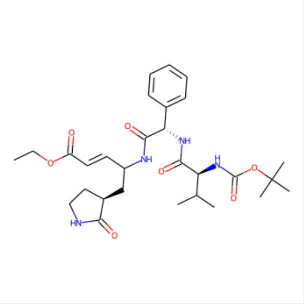	DB04692	Experimental	MPI6	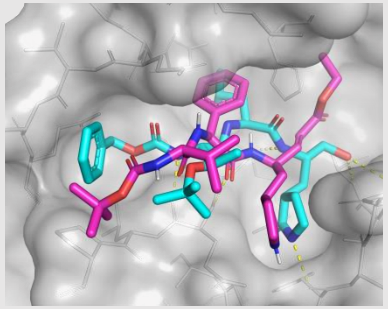

**23727975**	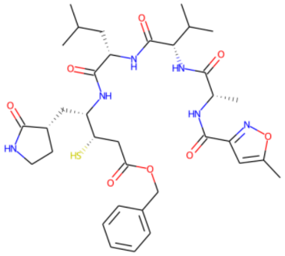	*NA*	Research	N3	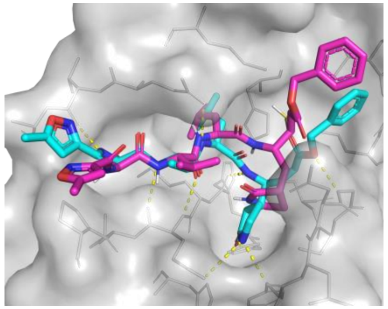
**14238620**	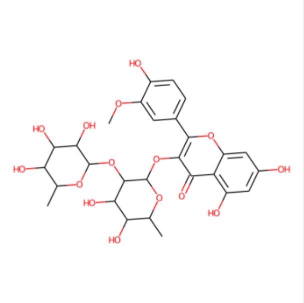	Calendoflaside	Research	Myricetin	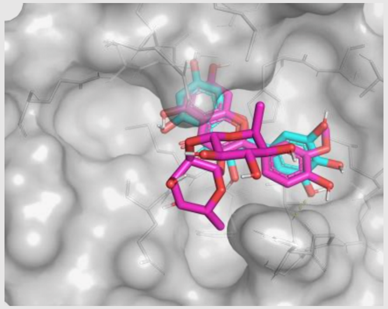
**5280343**	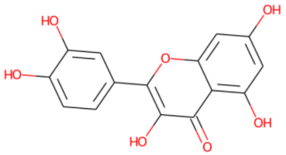	Quercetin	Experimental	Myricetin	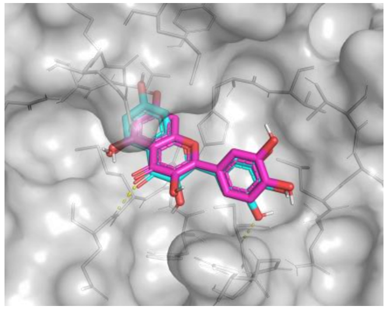

**5280863**	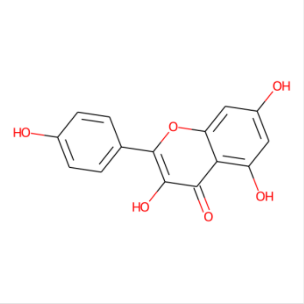	Kaempferol	Research	Myricetin	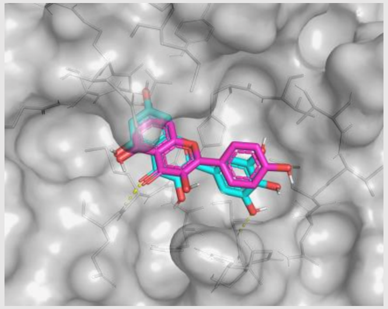
**5281616**	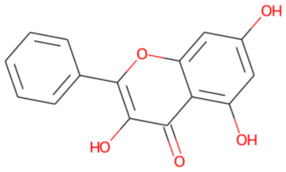	Galangin	Research	Myricetin	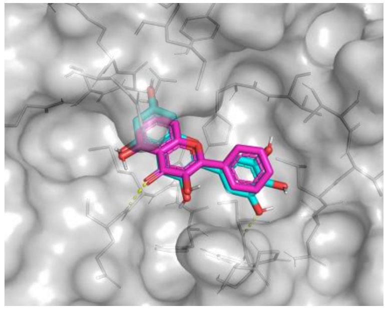
**5281673**	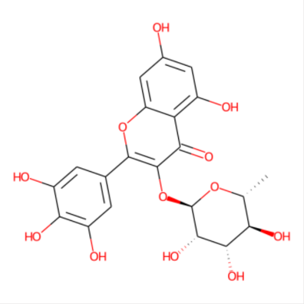	Myricitrin	Research	Myricetin	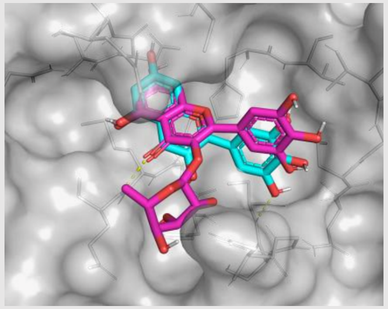

**5281692**	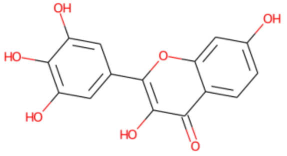	Robinetin	Research	Myricetin	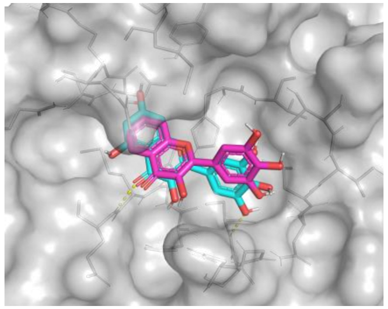
**5481963**	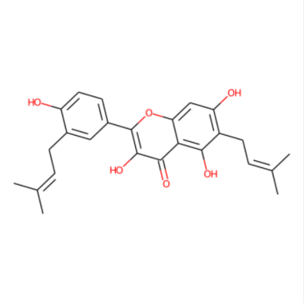	Glyasperin A	Research	Myricetin	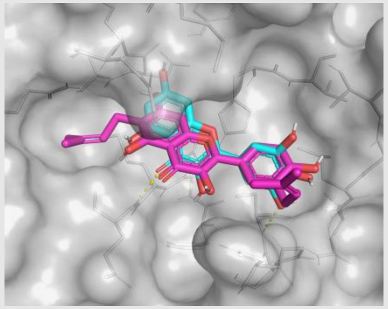
**6918652**	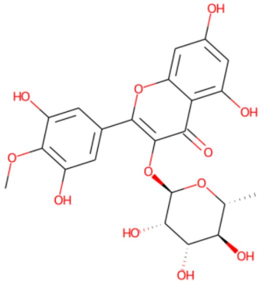	Mearnsitrin	Research	Myricetin	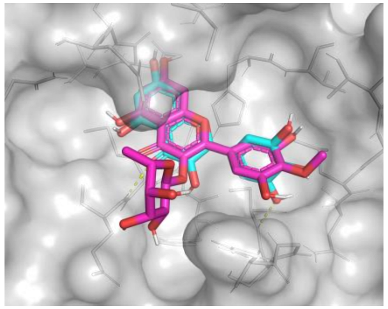

**9866908**	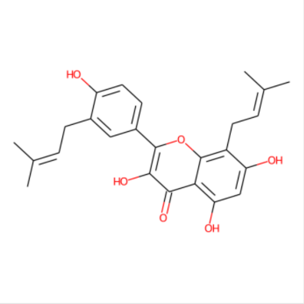	Broussoflavonol F	Research	Myricetin	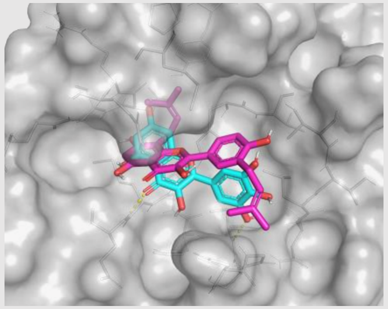
**5281691**	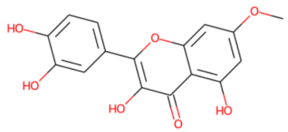	Rhamnetin	Research	7-O-methyl-myricetin	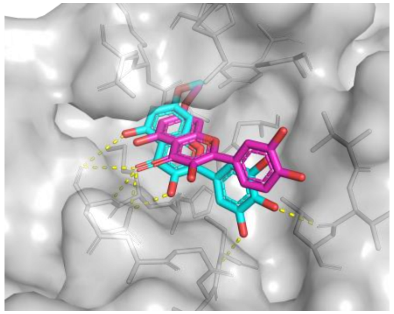
**712316**	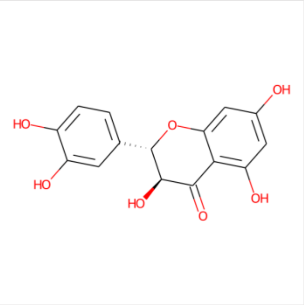	(-)-taxifolin	Research	7-O-methyl-dihydromyricetin	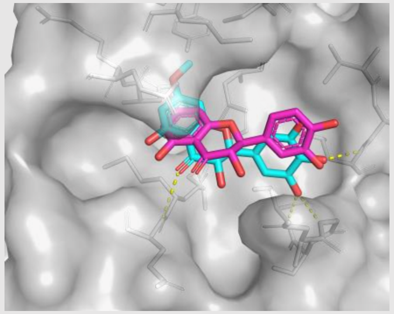

**5281607**	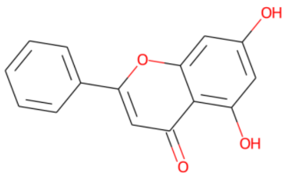	Chrysin	Experimental	Baicalein	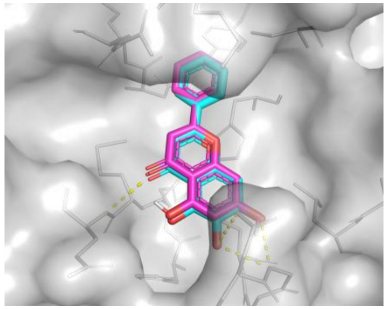
**5281697**	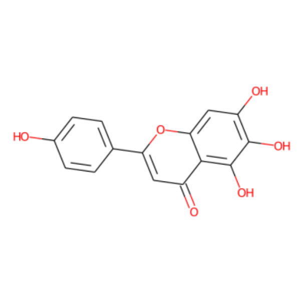	Scutellarein	Research	Baicalein	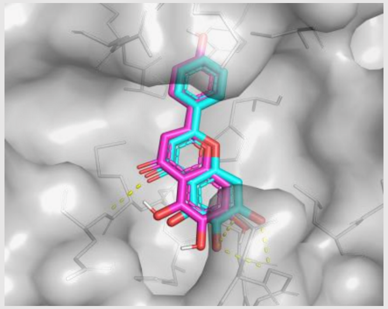
**64982**	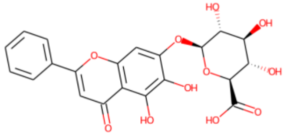	Baicalin	Research	Baicalein	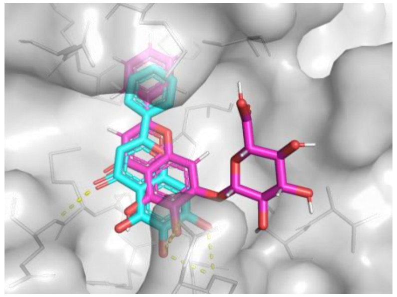

**9924495**	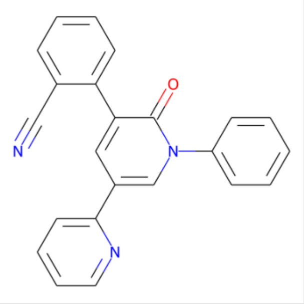	Perampanel	Approved	COMPOUND4	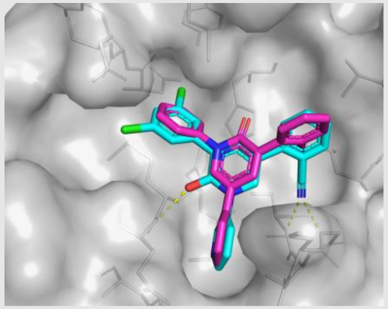
**644196**	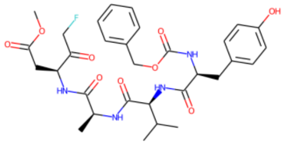	Caspase-1 Inhibitor VI	Research	Z-VAD(OMe)-FMK	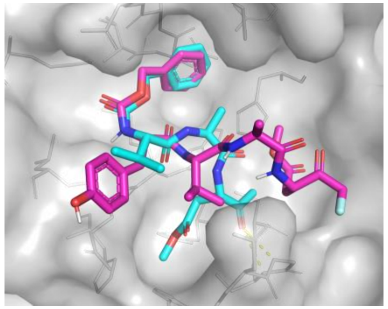

Note: The '**Inhibitor**' in the table is the most similar inhibitor for each CADD compound, and the Tanimoto similarity between the CADD compound and inhibitor is greater than 0.5.
