# Supplementary figures and images for: DrugDevCovid19: An Atlas of Anti-COVID-19 Compounds Derived by Computer-Aided Drug Design
Source: Molecules. 2022 Jan 21;27(3):683. doi: 10.3390/molecules27030683 (PMC8838031; doi:10.3390/molecules27030683)

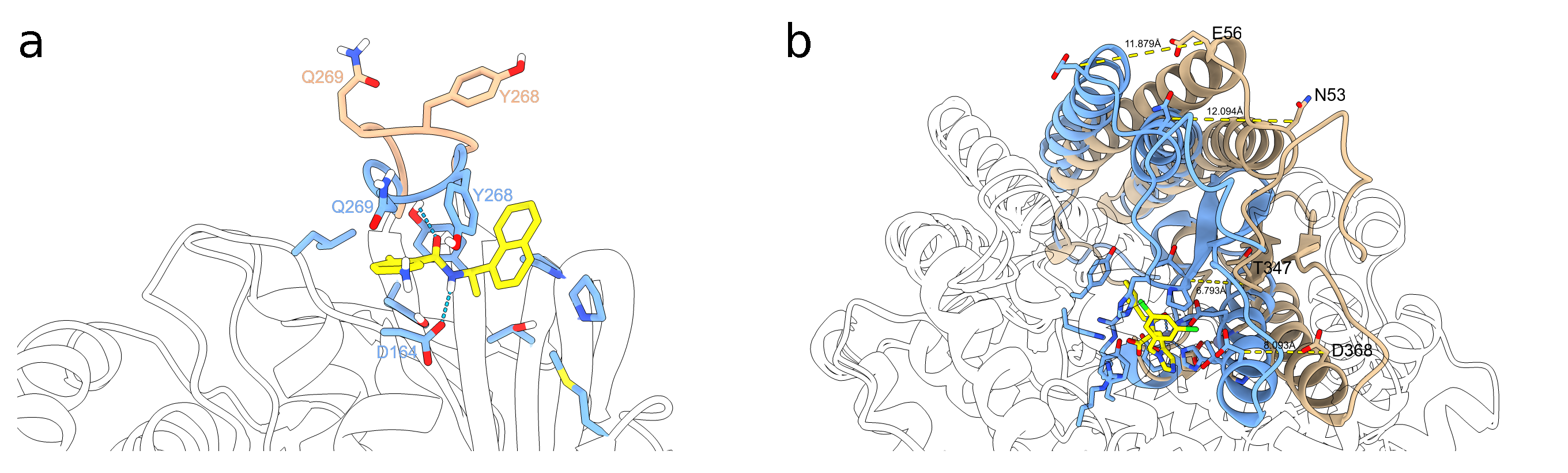

Supplement: Supplementary file 1 [file molecules-27-00683-s001.zip › Figure S1.tif]

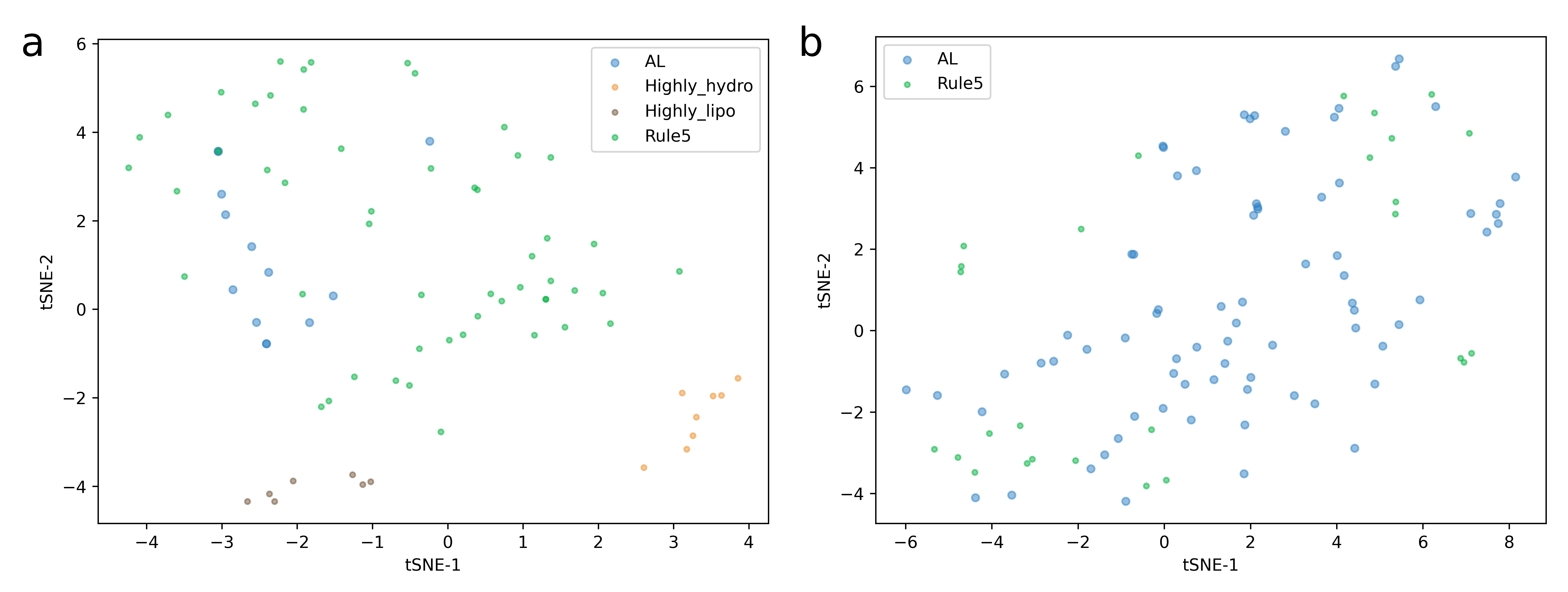

Supplement: Supplementary file 1 [file molecules-27-00683-s001.zip › Figure S2.tif]
